# Administration of Asian Herb Bennet (*Geum japonicum*) Extract Reverses Depressive-Like Behaviors in Mouse Model of Depression Induced by Corticosterone

**DOI:** 10.3390/nu11122841

**Published:** 2019-11-20

**Authors:** Dong Wook Lim, Taewon Han, Min Young Um, Minseok Yoon, Tae-Eun Kim, Yun Tai Kim, Daeseok Han, Jaekwang Lee, Chang Ho Lee

**Affiliations:** 1 Research Division of Functional Food Functionality, Korea Food Research Institute, Wanju 55365, Korea; dwlim@kfri.re.kr (D.W.L.); myum@kfri.re.kr (M.Y.U.); msyoon@kfri.re.kr (M.Y.); tekim@kfri.re.kr (T.-E.K.); ytkim@kfri.re.kr (Y.T.K.); imissu@kfri.re.kr (D.H.); jklee@kfri.re.kr (J.L.); 2 Food Functional Evaluation Support Team, Korea Food Research Institute, Wanju 55365, Korea; korea498@kfri.re.kr; 3 Division of Food Biotechnology, University of Science & Technology, Daejeon 34113, Korea

**Keywords:** *Geum japonicum*, antidepressant, neuroprotective, corticosterone, hypothalamic–pituitary–adrenal axis

## Abstract

*Geum japonicum*, commonly known as Asian herb bennet, has been used as a diuretic, astringent, anti-dizziness, and anti-headache agent in traditional medicine. Since the antidepressant-like effects of *G. japonicum* extract have not been well studied, we examined the antidepressant-like effects of *G. japonicum* extract using depressive-like behavior induced in mice through daily injection of corticosterone (CORT). ICR mice (male, 8 weeks old) were treated with CORT (40 mg/kg, i.p.) and orally administered using oral gavage needles with *G. japonicum* extract (30, 100, and 300 mg/kg) for 4 weeks. Behavioral experiments were performed 1 h after administration. The control mice exhibited a significant increase in the immobility times in the tail suspension and forced swim tests as well as the step-through latency time in the passive avoidance test. Further, the control group showed a significant decrease in their sucrose consumption. However, treatment with *G. japonicum* extract at doses of 100 and 300 mg/kg significantly improved these depression-like behaviors without altering the locomotor activity. Moreover, treatment with *G. japonicum* extract significantly prevented the decrease in the expression of brain-derived neurotrophic factor (BDNF) in the hippocampus. In addition, *G. japonicum* extract had neuroprotective effects against CORT-induced neurotoxicity in SH-SY5Y cells. Our study indicates that *G. japonicum* extract exhibits antidepressant-like activity in CORT-induced depressive mice, which might be as a result of increased BDNF expression.

## 1. Introduction

Depression is one of the most significant global health problems with an estimated more than 300 million patients worldwide [1]. However, currently available first-line antidepressants are not completely effective and usually cause a large number of undesirable side effects [2]. Thus, alternative approaches, including folk medicinal herbs, such as *Hypericum perforatum*, commonly known as St. John’s Wort [3], or increased consumption of antioxidants from fruits and vegetables [4,5] have garnered interest as potential antidepressant agents.

*Geum japonicum*, commonly known as Asian herb bennet, is used as a diuretic, astringent, anti-dizziness, and anti-headache agent in traditional Chinese medicine [6]. Previous studies have reported that *G. japonicum* crude extract and its active compounds such as triterpenoids have anticoagulant [7], antioxidant [8], anti-HIV [9], angiogenic [10], and vasorelaxant [11] activities. Ou et al. recently reported that *G. japonicum* extract has neuroprotective effects in the middle cerebral artery occlusion model via inhibition of inflammatory-mediated factors [12]. Nigaichigoside F1, isolated from *G. japonicum*, has been reported to alleviate hepatic steatosis in high-fat-diet-induced obese mice by regulating lipid metabolism genes [13]. The active fraction isolated from *G. japonicum* has also been reported to enhance capillary tube formation and promote the growth of new coronary collaterals in the ischemic region of the heart in the chronic coronary heart disease rat model [14]. However, the antidepressant-like effects of *G. japonicum* extract have not been well examined.

In this study, we aimed to investigate the antidepressant-like effects of *G. japonicum* extract in a depression animal model established by repeated chronic administration of corticosterone (CORT) injection. The CORT-induced depression model has been reported to be valid for evaluating the efficacy of potential antidepressants [15]. Further, we evaluated the neuroprotective effects of *G. japonicum* extract on CORT-induced neurotoxicity in human neuroblastoma SH-SY5Y cells.

## 2. Materials and Methods 

### 2.1. Preparation of the Standardized Geum japonicum

**Figure 1:**
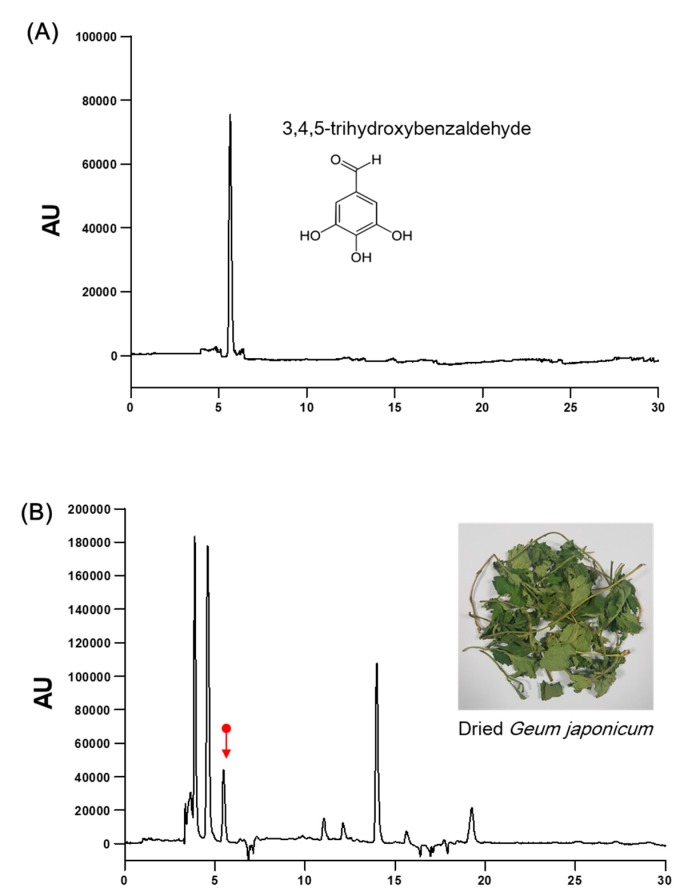
High-performance liquid chromatography (HPLC) chromatogram for standardization of *G. japonicum* extract. (**A**) HPLC chromatograms for 3, 4, 5-trihydroxybenzaldehyde and (**B**) *G. japonicum* extract. X-axis, retention time (min); Y-axis, absorbance unit (AU).

Dried *G. japonicum* (300 g) was extracted with 70% ethanol (3000 mL) at 80 °C for 6 h. The extract was filtered and lyophilized to dry powder. The yield of *G. japonicum* extract was 9.1%. The analysis of 3, 4, 5-trihydroxybenzaldehyde (3, 4, 5-THBA)(Sigma-Aldrich, MO, USA), an active or a standard compound [16] in *G. japonicum* extract, was performed on a high-performance liquid chromatography system (Jasco, Hachioji, Tokyo, Japan) equipped with a PU-980 pump, AS-950-10 autosampler, and MD-2010 PDA detector. An analytical chromatogram was obtained at 30 °C on a Waters Sunfire™ C18 5 μm column (4.6 mm × 250 mm) through gradient elution using a mobile phase composed of water, 0.2% (*v*/*v*) formic acid, and acetonitrile/MeOH (60:40, *v*/*v*). We performed gradient separation using 0.2% formic acid. The gradient was decreased by 90% from 0 to 10 min, 77% from 10 to 15 min, 40% from 15 to 35 min, and was increased by 90% from 35 to 40 min. The run time and flow rate were set at 40 min and 1.0 mL/min, respectively, while the samples were detected at 330 nm. The concentration of 3, 4, 5-THBA in extract powder was found to be 3.5 ± 0.12 mg/g using the peak area in the standard chromatogram (Figure 1).

### 2.2. Animals

All animal experiments were approved by the Institutional Animal Care and Use Committee of the Korea Food Research Institute (KFRI-M-19016). ICR mice (male, 7 weeks old, weighing 30–32 g) were purchased from KOATECH Animal Inc. (Pyeongteak, South Korea). The mice were kept at five mice per cage with free access to food and water prior to the experiments under controlled temperature (21 ± 2 °C) with a 24 h (12 h:12 h) light-dark cycle, (lights on at 07:00, and lights off at 19:00). The mice were acclimated at least 1 week prior to the experiments.

### 2.3. Corticosterone (CORT) and Extracts Administration

**Figure 2:**
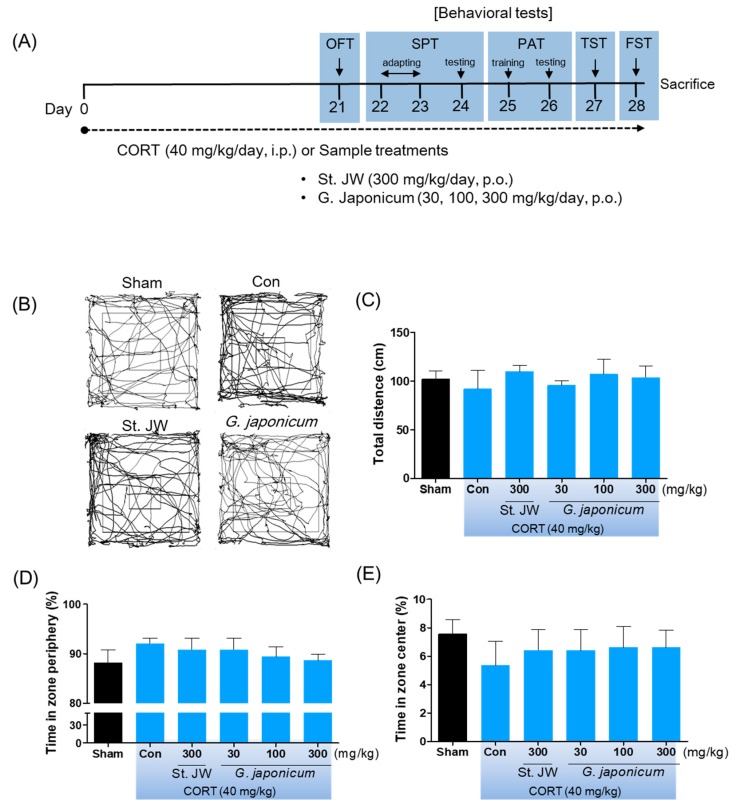
The effect of *G. japonicum* extract on the open field test in corticosterone (CORT)-induced depressive mice. (**A**) Study design of the experiments to evaluate the effect of *G. japonicum* extract. (**B**) Individual examples of locomotor activity are shown. (**C**) Total distance (cm) in traveled in OFT. (**D**) The number of line crossing in the periphery of the field. (**E**) The number of line crossing in the center of the field. There was no significant among-group difference in the locomotor activity. Results are presented as mean ± SEM.

CORT (Sigma-Aldrich, MO, USA) was dissolved in 0.9% (*w*/*v*) saline containing 1% Tween-80. *G. japonicum* extract or St. John’s wort (*Hypericum japonicum* 80% MeOH extract dried powder), which was the positive control, were dissolved in distilled water. The mice were randomly assigned to six groups of 10 mice each as follows: (1) the sham group; (2) control group; (3) St. John’s wort 300 mg/kg; (4) *G. japonicum* extract 30 mg/kg; (5) *G. japonicum* extract 100 mg/kg; and (6) *G. japonicum* extract 300 mg/kg treated group. Mice in the control group and the samples-treated groups were orally administered using gavage needles with received CORT (40 mg/kg, i.p.) injections once daily for 4 weeks, while those in the sham group were orally administered with distilled water and received intraperitoneal (i.p.) injections of an equal volume of vehicle. After 3 weeks of treatments, the mice underwent the depression-related behavioral tests beginning 1 hour after sample administration, as following the experimental scheme, for a week (Figure 2A) and were then sacrificed for western blots analysis.

### 2.4. Open Field Test (OFT)

After 3 weeks of treatment of *G. japonicum* extract or St. John’s wort, the OFT was performed as previously described [16]. To study locomotor activity, the mice were located in open maze (50 × 50 × 50 cm arena). Their locomotor-related behaviors recorded and measured the total distance and times in zone periphery and center for 5 min using SMART v3.0 software (Panlab SL, Barcelona, Spain).

### 2.5. Sucrose Preference Test (SPT)

The SPT was performed 24 hours after OFT, following a previously established protocol [17]. The two bottles contained the 1% sucrose solutions were located in the cage for 24 h. Next, one of the bottle of the sucrose solution was located with water for 24 h. The mice then were placed in cages individually with free access to the two bottles for 24 h, and the consumed volumes were recorded. Sucrose preference was calculated as follows: consumption (%) = [sucrose intake/(sucrose intake + water intake)] × 100.

### 2.6. Passive Avoidance Test (PAT)

The PAT was performed 24 hours after SPT, as in a previously described protocol for depression-related learning and memory [18]. The mice were tested in the passive-avoidance task (GEMINI, SD instruments, San Diego, CA, USA). In the training trial, each mouse was placed in the safe compartment with the closed door. After 1 min of acclimatization, the door opened and the mice were allowed to enter the dark compartment. When the mice entered the dark compartment, the mice received an inescapable electrical foot shock of 0.5 mA for 3 s. On the next day, the mice were again placed in the safe compartment, and the latency to enter the dark compartment was recorded.

### 2.7. Tail Suspension Test (TST)

To verify whether *G. japonicum* extract has antidepressant-like effects, the TST immobility was performed 24 hours after PAT. The mice were suspended by the tail using an adhesive tape attached to a hook connected to a strain gauge. The automated device (BioSeb, Chaville, France) was used to score immobility during the last 6 min.

### 2.8. Forced Swim Test (FST)

The FST immobility was performed 24 hours after TST. The mice were forced to swim by being individually placed in a clear Plexiglas cylinder (13 cm in height and 24 cm in diameter) filled with water (22–24 °C, 10 cm depth) for 6 min. The immobility time was determined during the last 4 min by SMART v3.0 software (Panlab SL, Barcelona, Spain).

### 2.9. Western Blotting

Mice were sacrificed by decapitation immediately after FST, the hippocampus was quickly isolated from brain. The hippocampus was homogenized in RIPA buffer containing protease inhibitor (Thermo Scientific, Waltham, MA, USA). The quantified proteins were separated by electrophoresis on 10% SDS-polyacrylamide gel electrophoresis (PAGE) and transferred onto polyvinylidene fluoride (PVDF) membranes (Millipore, Billerica, MA, USA). The membranes were blocked for 1 h at room temperature using 4% skimmed milk in Tris-buffer saline and probed overnight at 4 °C with primary antibodies raised against pro-BDNF (brain-derived neurotrophic factor) (1:1000 dilution, sc-65514, Santa Cruz Biotechnology, CA, USA). The membranes were incubated with the horseradish peroxidase-linked secondary antibody for 2 h. Then, immunoreactive proteins were detected by imaging systems; chemiluminescence (LI-COR Biosciences, Lincoln, NE, USA) and determined using the ImageJ software (NIH, Bethesda, MD, USA).

### 2.10. Cell Viability Assay

SH-SY5Y cells were cultured in Dulbecco’s Modified Eagle Medium (DMEM) (Gibco, NY, USA) containing 10% fetal bovine serum and 1% penicillin-streptomycin-glutamine. The cells were incubated in a humidified environment with 5% CO_2_ at 37 °C. The cell viability was determined by using the 3-(4, 5-dimethylthiazol-2-yl)-2, 5-diphenyltetrazolium bromide (MTT) assay. Briefly, the cells were seeded in 96-well plates (2 × 10^4^ cells/well) and co-treated with 30, 50, or 100 µg/mL of *G. japonicum* extract and CORT (200 μM) for 24 h. Next, MTT reagent (5 mg/mL) was added to the cell. After the plate incubation for 4 h, the medium was lysed and the formazan product was dissolved by DMSO. Cell viability was quantified by measuring the optical density at 570 nm using a microplate reader.

### 2.11. Statistical Analysis

Multiple between-group differences were compared using one-way analysis of variance followed by the Tukey’s post hoc test using Prism 5 (GraphPad Software, Inc., San Diego, CA, USA). All data were presented as mean ± standard error of the mean (SEM). Differences with *p* < 0.05 were considered significant.

## 3. Results

### 3.1. Effect of G. japonicum Extract on OFT

There was no significant among-group difference in the time spent in the center/periphery area and the total distance covered on the OFT (Figure 2B–E). Further, CORT injection did not have a significant effect on body weight (data not shown).

### 3.2. Effect of G. japonicum Extract on SPT

**Figure 3:**
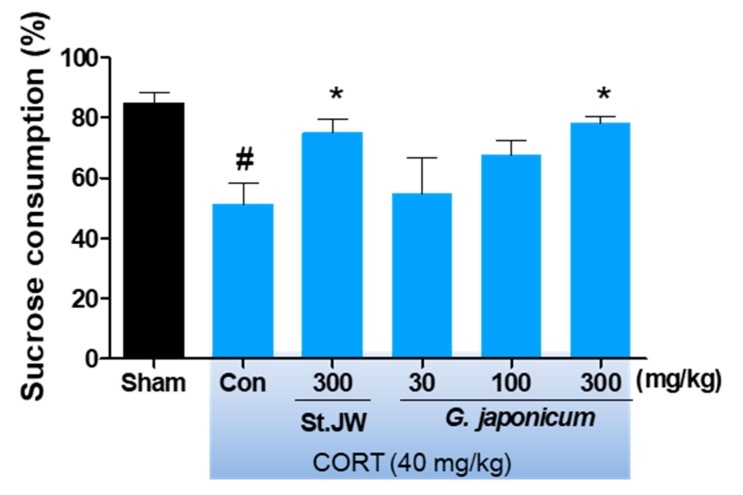
Effects of *G. japonicum* extract on sucrose consumption in CORT-induced depressive mice. CORT injections significantly decreased sucrose consumption, while treatment with 300 mg/kg *G. japonicum* extract significantly increased it. Results are presented as mean ± SEM. # *p* < 0.05 versus the sham group; * *p* < 0.05 versus the CORT-injected control group (Con).

The repeated CORT injection significantly decreased the percentage of sucrose consumption as compared with the sham group. However, *G. japonicum* extract at a dose of 300 mg/kg significantly increased the percentage of sucrose consumption (respectively, *p* < 0.05) (Figure 3).

### 3.3. Effect of G. japonicum Extract on PAT

**Figure 4:**
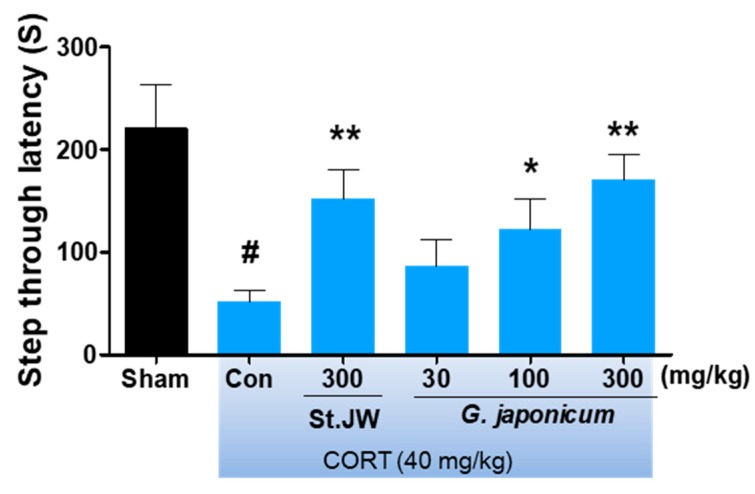
Effects of *G. japonicum* extract on the passive avoidance test in CORT-induced depressive mice. CORT injections significantly decreased step-through latency time (s), while treatment with 100 and 300 mg/kg *G. japonicum* extract significantly increased it. Results are presented as mean ± SEM. # *p* < 0.05 versus the sham group; ** *p* < 0.01 and * *p* < 0.05 versus the CORT-injected control group (Con).

As shown in Figure 4, the step-through latency of the CORT-induced depressive mice was significantly lower than that of the sham group; however, *G. japonicum* extract at a dose of 300 mg/kg significantly improved this deficit (*p* < 0.01).

### 3.4. Effect of G. japonicum Extract on TST and FST

**Figure 5:**
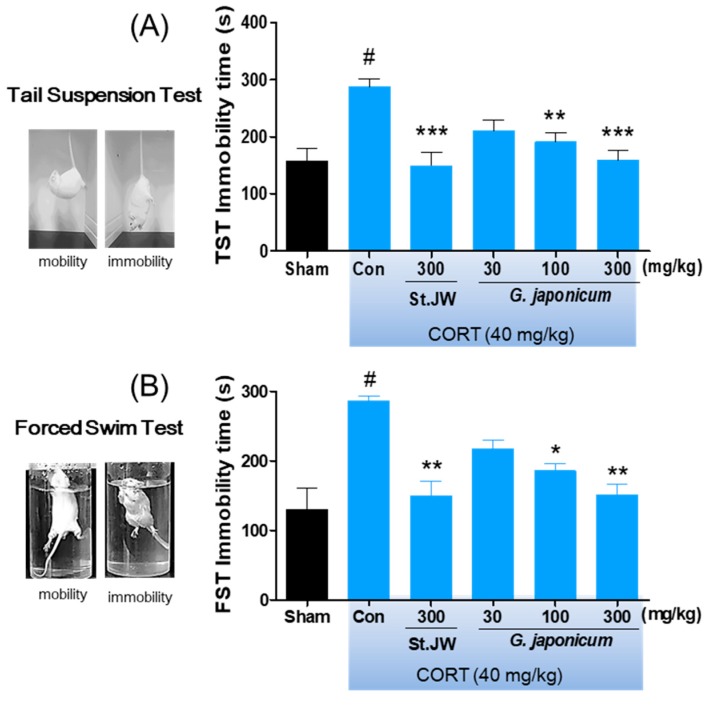
Effects of *G. japonicum* extract on the (**A**) tail suspension test (TST) and (**B**) forced swim test (FST) in CORT-induced depressive mice. CORT injections significantly increased the immobility time (s), while treatment with 100 and 300 mg/kg *G. japonicum* extract significantly decreased it. Results are presented as mean ± SEM. # *p* < 0.05 versus the sham group; *** *p* < 0.001, ** *p* < 0.01, and * *p* < 0.05 versus the CORT-injected control group (Con).

The repeated CORT injection significantly decreased the immobility times as compared with the sham group in the TST and FST. However, *G. japonicum* extract-treated groups had significantly reduced immobility times, with a maximum decrease in immobility of 32.1% at a dose of 300 mg/kg (Figure 5A,B).

### 3.5. Effect of G. japonicum Extract on BDNF Expression in the Hippocampus

**Figure 6:**
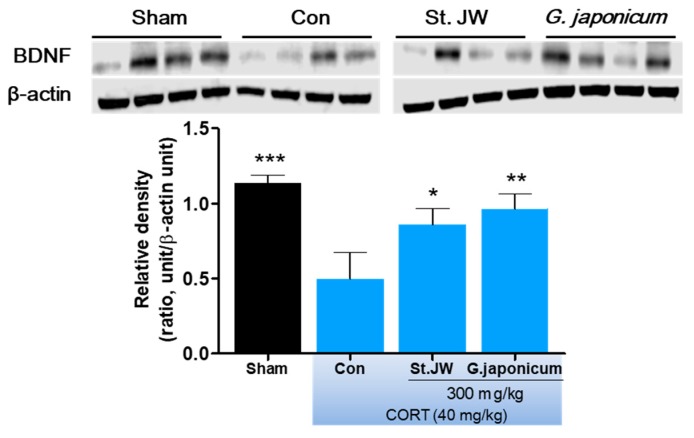
Effect of *G. japonicum* extract on hippocampal BDNF quantification by western blotting. CORT injections significantly decreased BDNF expression in the hippocampus, while treatment with 300 mg/kg *G. japonicum* extract significantly increased it. Results are presented as mean ± SEM. *** *p* < 0.001, ** *p* < 0.01, and * *p* < 0.05 versus the CORT-injected control group (Con). BDNF, brain-derived neurotrophic factor

After the FST behavior test, the mice hippocampus were isolated from whole brain for western blot analysis. As shown in Figure 6, repeated CORT (40 mg/kg, i.p.) injections for 28 days markedly reduced the BNDF protein expression levels in the hippocampus. However, *G. japonicum* extract 300 mg/kg and St. JW 300 mg/kg treated groups significantly prevented this deficit (*p* < 0.01).

### 3.6. Effect of G. japonicum Extract on Neurotoxicity

**Figure 7:**
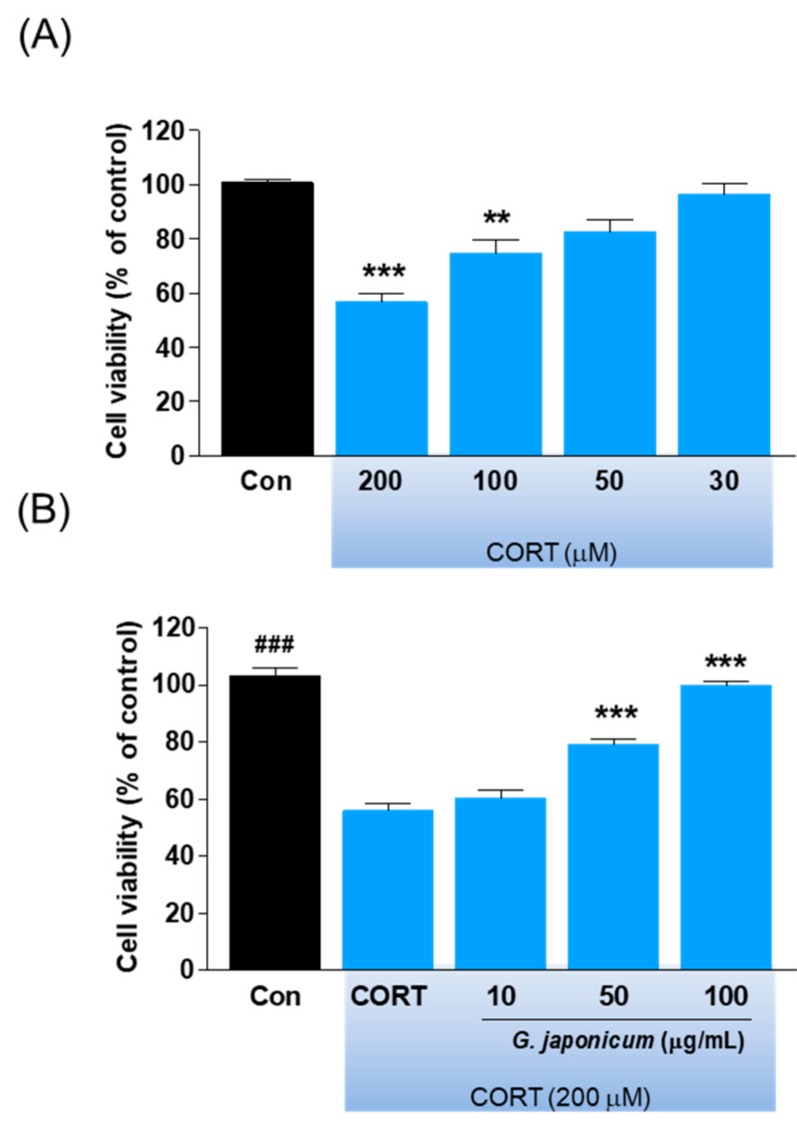
Effect of *G. japonicum* extract on CORT-induced cell viability in SH-SY5Y cells. (**A**) Dose-dependent cell death induced by CORT in SH-SY5Y human neuroblastoma cells. (**B**) *G. japonicum* extract showed neuroprotective effects against CORT-induced toxicity in SH-SY5Y cells. Results are presented as mean ± SEM. ### *p* < 0.001 versus the normal group; *** *p* < 0.001 and ** *p* < 0.01 versus the CORT-treated control group.

We determined the protective effects of *G. japonicum* extract on CORT-induced neurotoxicity in SH-SY5Y cells. We found that 200 μM CORT significantly decreased cell viability by 50% compared with the controls (*p* < 0.001; Figure 7A), which was prevented by co-treatment with *G. japonicum* extract (*p* < 0.001; Figure 7B).

## 4. Discussion

This is the first study on the antidepressant-like effects of *G. japonicum* extract on CORT-induced depression-like behaviors in mice, which is a well-established animal model for depression [19]. We found that *G. japonicum* extract prevented depressive-like behaviors, as shown by significantly decreased immobility times in the TST or FST and increased sucrose consumption without affecting locomotor activity. Moreover, *G. japonicum* extract improved cognitive function by prevention of reducing neurotrophic factor expression, which is involved in neuroplasticity. Additionally, *G. japonicum* extract showed protective effects against CORT-induced neurotoxicity in human neuroblastoma SH-SY5Y cells.

Although the complex pathogenesis of depression remains poorly understood, hypothalamic–pituitary-adrenal (HPA) axis dysfunction is well-accepted as a risk factor for stress-related disorders, such as depression or anxiety [20]. The association between HPA-axis dysfunction and depression is suggested by the fact that many depressed patients exhibit cortisol hypersecretion [21] and impaired glucocorticoid negative feedback system [22]. In addition, exposure to high cortisol levels might be injurious to the brain, especially in the hippocampus or frontal cortex, which have a high concentration of glucocorticoid receptors [23]. Given that depressed patients have been reported to have a smaller hippocampal volume compared with healthy controls [24], the hippocampal volume predicts antidepressant efficacy in depressed patients [25]. Similarly, HPA-axis dysfunction in in vivo models is modified by chronic stress and controlled by treatment with antidepressants [26,27]. Further, glucocorticoid overexposure by repeated injection of high doses of CORT has been implicated in hippocampal apoptosis [28] and depressive-like behaviors [29]. The state of immobility in the TST or FST is reported to mimic the depression phenotypes in humans and can be ameliorated by treatment with antidepressant drugs [30]. Sucrose consumption in animals has been used as an indicator of anhedonia-like behavior. Anhedonia is a core symptom of major depression in humans [31]. We found that mice injected daily with 40 mg/kg CORT showed significantly increased immobility times in the TST and FST as well as decreased sucrose consumption. Further, we found that they had shorter step-down latency in the passive avoidance test compared with non-injected normal mice. This is consistent with previous findings on CORT-induced depressive or memory deficit behaviors in rodents [32,33]. As expected, we found that *G. japonicum* extract-treated groups showed significantly reduced immobility times in the TST and FST without any changes of locomotor activity, especially at the dose of 300 mg/kg. Moreover, *G. japonicum* extract significantly increased the preference for sucrose consumption. These results suggest that *G. japonicum* extract has antidepressant-like effects in CORT-induced depression animal model.

BDNF, which is a member of the neurotrophins family, is highly expressed in the hippocampus and prefrontal cortex, and is involved in cognitive dysfunction and altered mood [34]. Numerous clinical studies have demonstrated that BDNF is an important factor in the pathogenesis of depression [35,36,37]. Further, serum BDNF levels are significantly increased by antidepressant treatment in depressed patients [38]. We found that the CORT-treated control group showed significantly decreased BDNF levels in the hippocampus. This is consistent with previously reported CORT-reduced BDNF expression in the hippocampus region [39]. As expected, we found that *G. japonicum* extract prevents the CORT-induced reduction in BDNF levels in the hippocampus. This suggests that *G. japonicum* extract potentially has antidepressant-like effects modulated by BDNF. However, the mechanism of the up-regulation of BDNF expression by *G. japonicum* extract remains unknown. Thus, further studies should confirm the antidepressant-like effects of *G. japonicum* extract using BDNF receptor, tropomyosin receptor kinase B (TrkB), and cyclic AMP response element-binding protein (CREB) signaling in the hippocampus or cerebral cortex.

CORT treatment has been reported to induce neuronal cell death by reducing BDNF expression in the hippocampus [40]. Further, glucocorticoid-induced cell injury has been used to screen potential antidepressants [41]. We used a model of neurotoxicity that mimics the glucocorticoid hypersecretion by exposing SH-SY5Y cells to CORT [17]. We found that SH-SY5Y cells co-treated with *G. japonicum* extract and CORT for 24 h showed reduced neurotoxicity in a concentration-dependent manner. This suggests that *G. japonicum* extract can protect against CORT-induced neurotoxicity.

A limitation of this study is that our in vivo findings present a portion of the animal’s behaviors results, and dose does not reveal the bioavailability of the active compounds of *G. japonicum* extract. Thus, active compounds of *G. japonicum* extract need to be tested in vivo for their efficacy. Moreover, further studies are necessary to determine whether active compounds from *G. japonicum* extract act on the central nerve system by blood-brain barrier penetration. Taken together, our results indicate that *G. japonicum* extract ameliorates CORT-induced depressive-like behaviors in mice and that this activity might be mediated by up-regulating BDNF expression. Further, *G. japonicum* extract resulted in neuroprotective effects against CORT-induced neurotoxicity in human neuroblastoma cells.

## 5. Conclusions

To our knowledge, this is the first study on the antidepressant-like effects of *G. japonicum* extract on CORT-induced depressive-like behaviors in mice. We found that *G. japonicum* extract prevents against CORT-induced depressive-like behavior and that this effect may be mediated by BDNF regulation, which is involved in inhibition of neuronal loss. Our results suggest that *G. japonicum* extract may be useful in the treatment of depression.
